# Combined Use of Ninjin'yoeito Improves Subjective Fatigue Caused by Lenalidomide in Patients With Multiple Myeloma: A Retrospective Study

**DOI:** 10.3389/fnut.2018.00072

**Published:** 2018-08-21

**Authors:** Tomoki Ito, Akiko Konishi, Yukie Tsubokura, Yoshiko Azuma, Masaaki Hotta, Hideaki Yoshimura, Takahisa Nakanishi, Shinya Fujita, Aya Nakaya, Atsushi Satake, Kazuyoshi Ishii, Shosaku Nomura

**Affiliations:** First Department of Internal Medicine, Kansai Medical University, Hirakata, Japan

**Keywords:** Chinese medicine, lenalidomide, multiple myeloma, fatigue, ninjin'yoeito

## Abstract

Lenalidomide is an immunomodulating derivative of thalidomide, which shows anti-tumor activity against myeloma cells with immunomodulation including augmentation of T-cell and natural killer cell function. Continuous treatment with this agent shows better survival benefit in patients with multiple myeloma and combined lenalidomide with dexamethasone (LEN-DEX) is a standard treatment regimen. However, fatigue is a frequent symptom resulting from lenalidomide administration. This side-effect therefore reduces quality of life for elderly patients and, furthermore, is a reason for treatment discontinuation. Unfortunately, appropriate preventive countermeasures against lenalidomide-related fatigue have not been established. Ninjin'yoeito is a traditional Chinese medicine made from the extracts of 12 herbal plants, which positively affects immunity and inflammation. It is used to treat fatigue, decreased appetite, anemia, and general malaise associated with malignant tumors and chemotherapy. We have previously reported that ninjin'yoeito significantly improved patients' subjective fatigue symptoms treated with melphalan-prednisone for multiple myeloma. In the present study, we assessed the benefits of ninjin'yoeito as a supplementary treatment for patients with myeloma, and its effect on lenalidomide treatment regime compliance. We retrospectively analyzed 36 cases of newly diagnosed or relapsed/refractory multiple myeloma. The study included patients receiving LEN-DEX with onset of general fatigue after lenalidomide administration (13 and 23 patients with or without ninjin'yoeito, respectively). Frequency of subjective fatigue was significantly decreased in patients administered ninjin'yoeito, compared to those treated with LEN-DEX alone (92.3 and 47.8 % of patients with and without ninjin'yoeito, respectively; *p* = 0.008). In addition, combined use of ninjin'yoeito and LEN-DEX showed a trend toward reduced rates of treatment discontinuation (7.7 % and 34.8 % of patients with and without ninjin'yoeito, respectively; *p* = 0.076). Our results suggest that ninjin'yoeito is an effective method for treating subjective fatigue caused by lenalidomide and may have the potential to extend lenalidomide treatment duration.

## Introduction

Lenalidomide (LEN) is an immunomodulating derivative of thalidomide and classified as immunomodulatory drug (IMiD) that has both direct tumoricidal and immunomodulatory effects in multiple myeloma (MM). LEN displays immunopotentiating activity, including augmentation of T and natural killer cell function (1–7).

LEN with dexamethasone (LEN-DEX) is a backbone regimen for MM. At present, the standard of care for patients with newly diagnosed or relapsed/refractory MM is to administer LEN combined with proteasome inhibitors or antibody-drugs. In addition, continuous treatment of LEN until progression diseases results in better survival benefit in MM patients (8–10).

Despite the benefits of LEN, general fatigue is sometimes an adverse event during its administration in clinical practice. In a randomized phase 3 trial consisting of newly diagnosed MM patients, grade 3 or 4 fatigue was observed in 7 to 9 % of the patients administered LEN-DEX as a first line treatment (9). Similarly, grade 3 or 4 fatigue has been reported for 6.5 % of relapsed or refractory MM patients (11). Furthermore, 47 % of patients receiving LEN maintenance therapy after autologous stem-cell transplantation experience fatigue to some extent (8). In clinical practice, LEN dosage is often reduced or treatment discontinued as a result of patients experiencing fatigue in addition to dysgeusia, anemia, and gastrointestinal symptoms. Unfortunately, at present there is no effective countermeasure against these adverse events.

Ninjin'yoeito (NYT) is a traditional Chinese medicine made from the extracts of 12 herbal plants, which has known beneficial effects on immunity and inflammation (12, 13). Components of NYT is listed in STORK (http://mpdb.nibiohn.go.jp/stork/). NYT has been used to treat fatigue, anorexia, anemia, and coldness of hands and feet. Notably, NYT not only has a supportive effect against MM with treatment of melphalan, but also relieves subjective symptoms, such as fatigue and pain (14). Although its mechanism of action is still largely unknown, NYT is therefore expected to improve quality of life for MM patients. In the present study, we focused on these activities and assessed whether combination of NYT is useful against the LEN-related subjective fatigue. Furthermore, we investigated whether discontinuation of LEN can be improved by combination use of NYT.

## Patients and methods

### Patients and treatment

We retrospectively analyzed 36 cases of newly diagnosed or relapsed/refractory receiving LEN-DEX with (*n* = 13) or without (*n* = 23) NYT between January 2011 and December 2017, who experienced general fatigue as a result of LEN-DEX treatment (Table 1). Patients received either oral lenalidomide 5–25 mg per day (depending on the judgment of a doctor in charge) on days 1–21 of each 28–day cycle. All patients received 20–40 mg oral dexamethasone on days 1, 8, 15, and 22 of each 28–day cycle (for 4 cycles) until disease progression. After 1 cycle-treatment of LEN-DEX, the severity of fatigue was graded using the National Cancer Institute Common Terminology Criteria for Adverse Events (NCI CTCAE) version 4.0 (code 10016256, grade 1-3) by clinical interview. We started oral administration of NYT (Kracie Pharma, Ltd) at a dose of 5.0 g/day when patients complained of grade 1 or more fatigue after LEN-DEX treatment according to the patients' requests, while continued LEN-DEX alone if the patients did not want NYT. Patient base line characteristics are shown in Table 1. We assessed the effectiveness of NYT for the severity or grade of subjective fatigue by clinical interview after every treatment cycle and we collected the data of discontinuation of LEN-DEX up to 6 months after administration. LEN-DEX were discontinued in total 9 patients. In these cases, severity or grade of fatigue was evaluated at the last cycle of LEN-DEX administration.

**Table 1:** Patient base line characteristics.

	**Lenalidomide + NYT (*n* = 13)**	**Lenalidomide alone (*n* = 23)**	***p*-value**
**Age, median (range), years**	72 (53-85)	67 (45-79)	*p* = 0.213
**Sex, male/female, %**	38.5 / 61.5	47.8% / 52.2%	*p* = 0.731
**ECOG PS score**, ***n*** **(%)** 0 1 2	5 (38.5) 5 (38.5) 3 (23.1)	11 (47.8) 8 (34.8) 4 (17.4)	*p* = 0.847
**ISS stage**, ***n*** **(%)** I II III	4 (31.0) 6 (46.2) 3 (23.1)	6 (26.1) 9 (39.1) 8 (34.8)	*p* = 0.765
**No. of prior therapies**, ***n*** **(%)** 0 1 ≧2	2 (15.4) 5 (38.5) 6 (46.2)	7 (30.4) 8 (34.8) 8 (34.8)	*p* = 0.589
**M protein subtype**, ***n*** **(%)** IgG IgA BJP	7 (53.8) 3 (23.1) 3 (23.1)	14 (60.9) 4 (17.4) 5 (21.7)	*p* = 0.898
**Doses of LEN, mean** ± **SD**	14.2 ± 3.4 mg	14.6 ± 4.5 mg	*p* = 0.804
**Severity of fatigue**, ***n*** (%)^*^ Grade 1 Grade 2 Grade 3	8 (61.5) 4 (31.0) 1 (7.7)	17 (73.9) 5 (21.7) 2 (8.7)	*p* = 0.797

ECOG, Eastern Cooperative Oncology Group; PS, Performance Status; ISS, International staging system; BJP, Bence Jones protein.

* Severity of fatigue was graded after 1 cycle-treatment of LEN-DEX and before administration of NYT.

*P-value regarding “age” was calculated by unpaired t-test with Welch's correction. P-value regarding “sex” was calculated by a two-sided Fisher's exact-test. P-value regarding “PS score,” “ISS stage,” “prior therapies,” “M protein subtype,” “doses of LEN,” and “Severity of fatigue” were calculated by a Chi-square test*.

This study was carried out in accordance with the recommendations of the International Conference on Harmonization guidelines for Good Clinical Practice with written informed consent from all subjects. All subjects gave written informed consent in accordance with the Declaration of Helsinki. The protocol was approved by the Institutional Review Board of Kansai Medical University.

### Statistical analysis

The data from 2 groups (LEN-DEX with and without NYT) were compared by 2-way Contingency Table Analysis using a Fisher's exact test. For the background of patient profile, *p*-value regarding “age” and “sex” were calculated by unpaired *t*-test with Welch's correction and two-sided Fisher's exact test, respectively. *P*-value regarding “PS score”, “ISS stage,” “prior therapies,” “M protein subtype,” “doses of LEN,” and “Severity of fatigue” were calculated by a Chi-square test. A value of *p* < 0.05 was considered statistically significant. Data analysis was carried out with the software GraphPad Prism.

## Results

**Figure 1:**
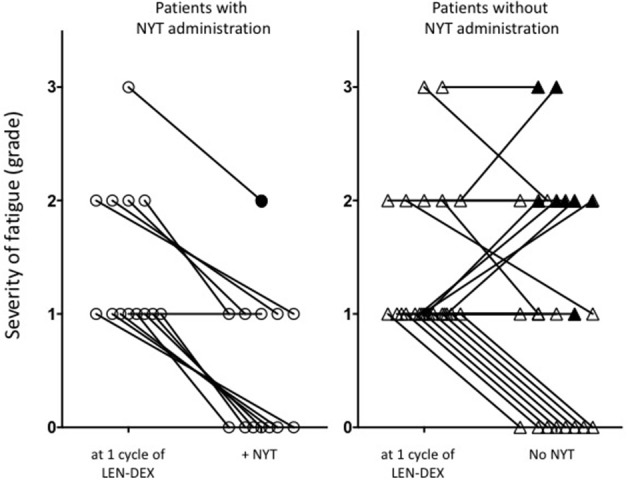
Change of fatigue grade in patients treated with or without NYT. After 1 cycle-treatment of LEN-DEX, the severity of fatigue was graded, and then NYT was administered according to the patients' requests. When the patients did not want NYT, LEN-DEX alone were continued. Assessment of the effectiveness of NYT for the severity of subjective fatigue and LEN-DEX discontinuation up to 6 months. ○, Patients with NYT administration; Δ, Patients without NYT administration; ● and ▴, show LEN-DEX-discontinued patients within 6 months. For these 9 discontinued patients, severity of fatigue was evaluated at the last cycle of LEN-DEX administration.

NYT was administered to 13 of the 36 patients with onset of general fatigue after 1 cycle of lenalidomide administration. Severity of fatigue in patients was shown in Figure 1 and Table 1. For 12 patients (92.3 %), the grade of fatigue was reduced by the use of NYT in conjunction with LEN-DEX (Figure 1 and Table 2). Of the 23 patients who continued LEN-DEX without NYT, 11 (47.8 %) had decrease in fatigue; however, the remaining 12 saw no improvement (5 had increased grade in fatigue during treatment) (Figure 1). Hence, NYT resulted in a significant increase in frequency of lower fatigue grade following its administration (*p* = 0.008).

**Table 2:** Frequency of reduced grade of fatigue during treatment.

	**Improvement of grade in fatigue**	**No improvement in fatigue**	***p*-value**
LEN-DEX + NYT (*n* = 13)	12	1	*p* = 0.008
LEN-DEX alone (*n* = 23)	11	12	

*The data from 2 groups were analyzed by 2-way Contingency Table Analysis with the use of a one-sided Fisher's exact test*.

In addition, 12 of the 13 patients (92.3 %) administered NYT were able to continue LEN-DEX at least for the 6 months (Figure 1 and Table 3). Of the 23 patients who did not receive NYT, 8 (34.8 %) failed to continue LEN-DEX. Hence, combined use of NYT and lenalidomide had a tendency toward suppressing LEN-DEX treatment discontinuation (7.7 and 34.8 % of patients with and without NYT, respectively, were unable to continue LEN-DEX treatment; *p* = 0.076).

**Table 3:** Continuation of LEN-DEX treatment.

	**Continuation of LEN-DEX**	**Discontinuation of LEN-DEX**	***p*-value**
LEN-DEX + NYT (*n* = 13)	12	1	*p* = 0.076
LEN-DEX alone (*n* = 23)	15	8	

*The data from 2 groups were analyzed by 2-way Contingency Table Analysis with the use of a one-sided Fisher's exact test*.

## Discussion

MM remains an incurable disease with a severe prognosis; it is therefore necessary to aim for long-term disease control by specific and effective drugs. It was demonstrated that continuous therapy prolongs progression-free survival until first and second relapse and overall survival (15). Currently, lenalidomide is the main drug prescribed for continuous and maintenance therapy (16). After stem-cell transplantation, lenalidomide maintenance significantly prolonged not only progression-free survival, but also overall survival (8, 10). Therefore, avoiding dose reduction or treatment discontinuation due to intolerance and side-effects is important for extending patient survival and improving quality of life. The most common adverse events associated with lenalidomide include hematologic events, such as neutropenia, anemia, and thrombocytopenia; gastrointestinal disorders, such as nausea, vomiting, constipation, and diarrhea; and other side-effects, such as infections, and skin rashes. General disorders, such as fatigue and pyrexia are also significant adverse events (8, 9), which cause difficulty in continuing lenalidomide maintaining treatment regimens in clinical practice. However, there is no effective means for treating these general and subjective symptoms, with the exception of dose reduction or treatment discontinuation.

Previously, we found that NYT had the potential to repress general fatigue caused by melphalan (14). The present study suggests that NYT may also be useful for improving the symptoms of subjective fatigue caused by lenalidomide in patients with MM. A possible mechanism of NYT to relieve the fatigue caused by chemotherapy could be an effect of *Schisandra* as components of NYT. *Schisandra* has been shown to possess anti-athletic fatigue activity in mice (17) and improve endurance and energy metabolism in exercised rats (18). *Schisandra* has a potential to upregulate peroxisome proliferator-activated receptor-gamma coactivator (PGC)-1alpha in skeletal muscle, which is a key regulator of energy metabolism (18). Further studies are needed to clarify the detailed mechanisms involved in the anti-fatigue properties of NYT.

Supplementing lenalidomide administration with NYT could result in increased treatment durations and hence increase life expectancy. The limitations of our report are the retrospective analysis and a small number of patients derived from the setting of everyday clinical practice including several biases. In addition to this, there is a possibility that the effect we observed are attributed to the placebo effect. Additional research examining clinical outcomes using NYT need to be studied in the future.

## Author contributions

TI planned, designed and wrote the paper. AK, YT, YA, MH, HY, TN, SF, AN, AS, KI, and SN contributed to acquisition and collection of patients' data. All authors read and approved the final manuscript.

### Conflict of interest statement

The authors declare that the research was conducted in the absence of any commercial or financial relationships that could be construed as a potential conflict of interest.
